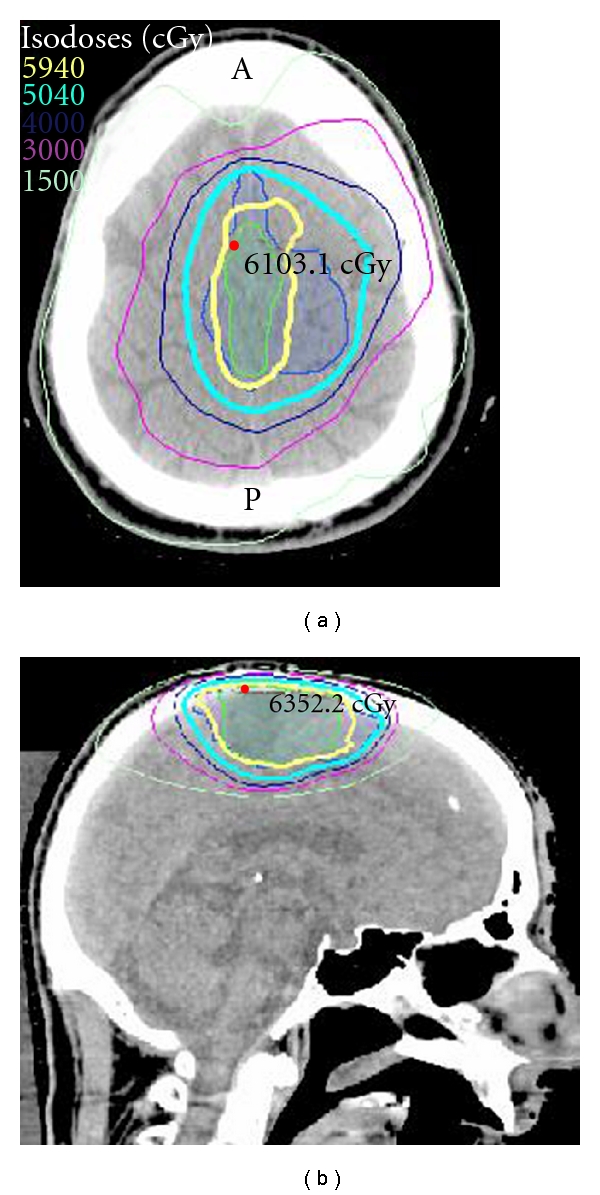# Primary Meningeal Rhabdomyosarcoma

**DOI:** 10.1155/2011/312802

**Published:** 2011-06-23

**Authors:** Manisha Palta, Richard F. Riedel, James J. Vredenburgh, Thomas J. Cummings, Scott Green, Zheng Chang, John P. Kirkpatrick

**Affiliations:** ^1^Department of Radiation Oncology, Duke University Medical Center, Durham, NC 27710, USA; ^2^Division of Medical Oncology, Department of Medicine, Duke University Medical Center, Durham, NC 27710, USA; ^3^Department of Pathology, Duke University Medical Center, Durham, NC 27710, USA

## Abstract

Primary meningeal rhabdomyosarcoma is a rare primary brain malignancy, with scant case reports. While most reports of primary intracranial rhabdomyosarcoma occur in pediatric patients, a handful of cases in adult patients have been reported in the medical literature. We report the case of a 44-year-old male who developed primary meningeal rhabdomyosarcoma. After developing episodes of right lower extremity weakness, word finding difficulty, and headaches, a brain magnetic resonance imaging (MRI) demonstrated a vertex lesion with radiographic appearance of a meningeal-derived tumor. Subtotal surgical resection was performed due to sagittal sinus invasion and initial pathology was interpreted as an anaplastic meningioma. Re-review of pathology demonstrated rhabdomyosarcoma negative for alveolar translocation t(2;13). Staging studies revealed no evidence of disseminated disease. He was treated with stereotactic radiotherapy with concurrent temozolamide to be followed by vincristine, actinomycin-D, and cyclophosphamide (VAC) systemic therapy.

## 1. Case Presentation

**Figure 1:**
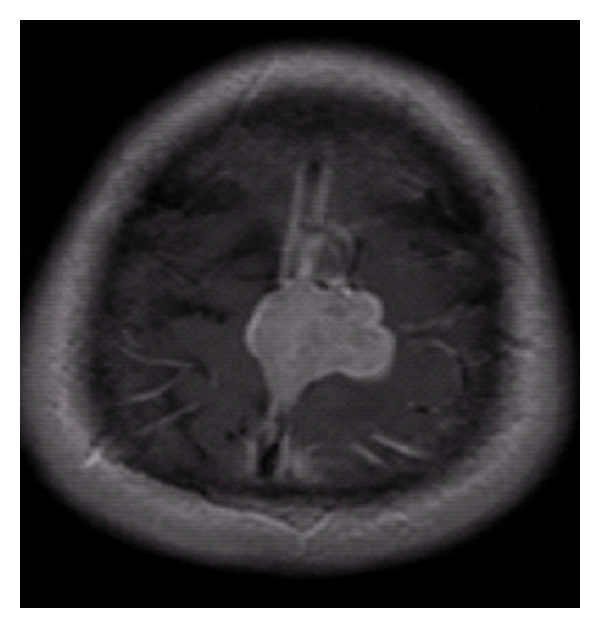


The patient was a 44-year-old Caucasian male with no significant past medical history who initially experienced episodes of right lower extremity weakness lasting 2-3 minutes that first occurred in June 2010. He had 2 such episodes in early June and within a few weeks was experiencing 5-6 episodes weekly. Shortly thereafter he noticed impairment in fine motor skills of the right leg with inability to put on shoes, word-finding difficulty, and severe headaches that awoke him from sleep. He was seen by his primary care physician, and MRI demonstrated a 2.7 × 2.5 × 3.1 cm brain lesion arising from the meninges (Figure 1). He was informed of the imaging results and the following day developed a seizure that started focally in his right arm and leg before becoming generalized. 

He was evaluated in the emergency room and admitted to the hospital for further evaluation and management. Surgical resection was performed July 2010 with pathology initially interpreted as an anaplastic meningioma. Complete resection was not possible given extensive invasion into the sagittal sinus. Postoperatively, the patient reported right-sided motor impairment that improved dramatically with physical therapy. His headaches and word-finding difficulty resolved completely postoperatively, and he was discharged from the hospital on postoperative day no.3. A brain MRI obtained 10 days following surgery showed a left frontoparietal vertex resection cavity measuring 2 × 1.5 cm with enhancement anteriorly and posteriorly. 

**Figure 2:**
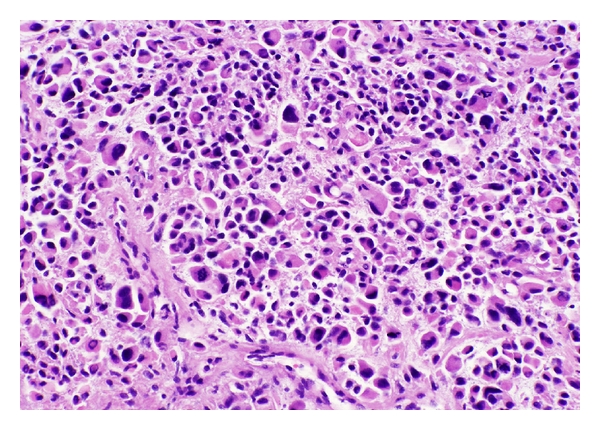


**Figure 3:**
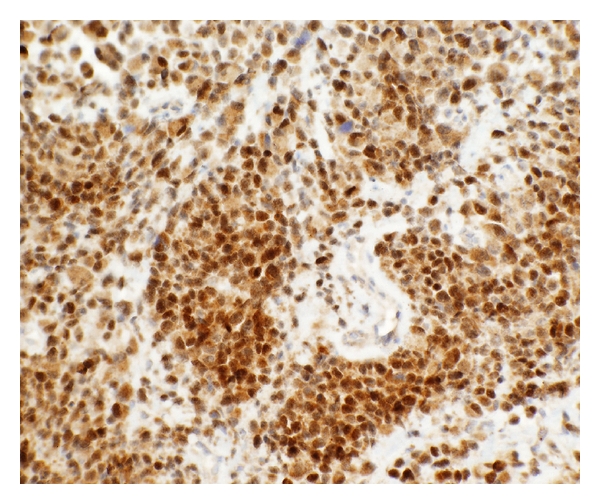


**Figure 4:**
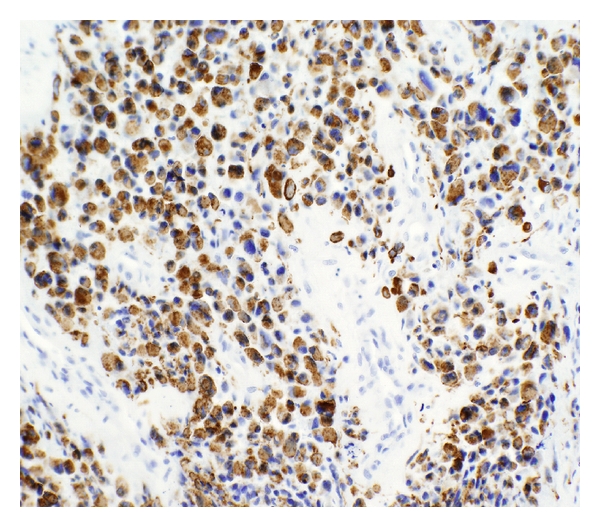


He presented to our institution for an opinion regarding further therapy. On exam, he was noted to have right upper and lower extremity weakness and wide-based gait with right foot drop. Pending review of pathology, we tentatively recommended highly conformal radiotherapy. Pathologic review confirmed a high-grade sarcoma consistent with rhabdomyosarcoma (Figure 2). Tumor cells were found to express myoD1 (Figure 3), desmin (Figure 4), and myoglobin. There was no evidence of meningothelial, glial, or neuronal component. Fluorescence *in situ* hybridization was performed, and the typical gene rearrangement (t2:13) seen in alveolar rhabdomyosarcoma was not visualized. Additional staging studies, including PET imaging, spine MRI, bone marrow biopsy, and lumbar puncture, were obtained, showing no evidence of metastatic disease.

After generating a variety of radiotherapy plans, a volumetric modulated arc therapy (VMAT) plan utilizing a Novalis Tx linear accelerator system (Varian Palo Alto, Calif, USA and BrainLAB, Munich, Germany) was ultimately selected in order to maximize target coverage while minimizing normal tissue toxicity. In addition, we offered concurrent temozolomide as a radiosensitizer [1]. The primary target volume encompassed contrast-enhancing tumor on T1-weighted preoperative MRI, uniformly expanded by 5 mm and further customized to extend 2-3 mm further along the meninges in all directions. The boost volume included the resection cavity, including areas of anterior and posterior postoperative enhancement on MRI, with 3 mm expansion and was customized to extend along the meninges in all directions. We prescribed 5040 cGy in 180 cGy fractions to the primary target volume followed by an additional 900 cGy boost in 180 cGy fractions to a total dose of 5940 cGy. The blue- and green-shaded volumes represent the primary and boost volumes, respectively. The thickened yellow and cyan lines signify the 5040 cGy and 5940 cGy isodose lines, respectively (Figure 5). Critical normal tissues, including the brainstem, optic chiasm, optic nerves, and eyes all received minimal dose. 

The patient tolerated chemoradiation well. Aside from mild fatigue, he developed headaches during treatment which improved with a low dose of steroids that was gradually tapered. Blood counts remained stable throughout radiotherapy. At the conclusion of chemoradiotherapy, the patient began systemic therapy, consisting of vincristine, actinomycin-D, and cyclophosphamide (VAC) per standard treatment for rhabdomyosarcoma. 

## 2. Literature Review

Rhabdomyosarcoma, the most common pediatric soft tissue sarcoma, has a predilection for the head and neck, genitourinary organs, and extremities [2]. The mean incidence of primary brain sarcomas is roughly 3% [3]. Among these few primary brain sarcomas, 70% arise in the pediatric population and there have been limited cases reported in adults [4]. These small numbers may be due, in part, to histological and anatomic uncertainty. Meningeal rhabdomyosarcomas must be distinguished from medullomyoblastoma, gliosarcoma, and teratomatous germ cell tumors all of which can have rhabdomyoblastic differentiation [5]. Although the meninges are composed of both mesenchymal and neuroectodermal cell lineages, it is the pluripotent mesenchymal cells that appear to give rise to meningeal rhabdomyosarcomas. This is in contrast to intracranial rhabdomyosarcomas which more likely arise from the brain substance and extend to the meninges [6]. 

Staging with lumbar puncture and imaging is important for identification of all sites of disease. Lumbar puncture or imaging of the craniospinal axis can identify leptomeningeal or multifocal disease which can have implications on radiotherapy fields and total dose. Few cases have reported diffuse meningeal involvement, but it is associated with a poorer prognosis [7, 8]. In addition, an extracranial primary site should be excluded with CT or PET imaging. 

Once histological diagnosis and staging is preformed, there is still much debate regarding appropriate further treatment. A review of published literature identified 38 previously reported cases of intracranial rhabdomyosarcoma with a mean survival of 9.1 months [9]. In this series, most cases were managed with surgical resection, when feasible, and 22 of the 38 patients received adjuvant radiotherapy with total dose range from 2500 to 8200 cGy. In cases of localized disease, extrapolation of data from the International Rhabdomyosarcoma Study Group (IRSG) has been applied [10]. In this study of parameningeal rhabdomyosarcoma, it was recommended that radiotherapy be delivered to the primary site, the adjacent meninges, and region of intracranial extension with an appropriate margin, omitting treatment to the entire brain or spinal axis which is associated with heightened toxicity. 

The inherent difficulty in discerning the cell of origin in rhabdomyosarcoma makes the selection of appropriate chemotherapy difficult and usefulness debatable. In the review by Celli et al., chemotherapy was administered in 11 of 38 cases reported but given heterogeneity of treatments little could be determined regarding relative effectiveness. Recent case reports have implemented the use of VAC chemotherapy which is standard in the treatment of systemic rhabdomyosarcoma [11]. 

Our patient represents one of the few adults with primary meningeal rhabdomyosarcoma. Given the rarity of these tumors, a multidisciplinary approach is imperative. While maximal safe surgical resection followed by adjuvant radiotherapy and chemotherapy is a logical approach to maximize long-term survival, further study is required to identify an optimal therapeutic strategy.

## Figures and Tables

**Figure 5:**